# Epidemiological factors affecting health service utilization in diabetic patients in Ethiopia

**DOI:** 10.1093/eurpub/ckac131.114

**Published:** 2022-10-25

**Authors:** R Benoni, A Sartorello, E Paiola, G Andreani, F Moretti, A Tsegaye, S Tardivo, F Manenti

**Affiliations:** 1 Department of Diagnostics and Public Health, University of Verona, Verona, Italy; 2 Doctors with Africa CUAMM, Padua, Italy

## Abstract

Diabetes-related deaths reached 2 million in 2019. The highest percentage of undiagnosed diabetes (59.7%) was observed in Africa, where accessibility to health services is pivotal to improving the outcome of diabetic patients. The study aims to assess the association between diabetic patients’ epidemiological factors and accessibility to healthcare services in a low-income country. The retrospective cohort study included diabetes-related outpatient department (OPD) visits and hospitalizations from 01/01/2018 to 31/08/2021 at St Luke Hospital (Ethiopia). Potential predictors were sociodemographic factors, COVID-19 cases, mean monthly temperature, and precipitations. The ARIMA method was applied to OPD visits and hospitalizations time series. OPD visits increased over time (p < 0.001) while hospitalizations were stable. The time series model was ARIMA(0,1,1) for OPD visits and ARIMA(0,0,0) for hospitalizations. Diabetes OPD patients were 1,685 (F = 732, 43%). Females had an average of 16% fewer OPD accesses per month (p = 0.002). Patients missing follow-up were 801 (48%). The time between follow-ups was longer as age increased (p < 0.001). There were 57 fewer forecast OPD visits per month on average using COVID-19 cases as ARIMA regressor. OPD visits decreased differently by geographic area as COVID-19 cases increased (p < 0.001). Hospitalized patients for diabetes were 408, 85 (20.8%) newly diagnosed. The odds ratio (OR) of diagnosis at admission was lower as age increased (OR 0.98, p = 0.009). Compared to type 1 diabetes, hospitalized females with type 2 (117-39.7%) were fewer than males (p = 0.019). Readmissions were 52, 10 (19.2%) within 30 days, without OR difference by sex, age, or diabetes type. Despite an increase in OPD visits for diabetic patients over the study period, the number of losses at follow-up and diagnoses at hospitalization remains high. Gender and age influenced service utilization. Females’ access to care is still problematic (concept of “missing women”).

**Key messages:**

• Primary health care should be implemented to improve access to health services and diabetes management.

• Ensuring equity in healthcare accessibility should be a priority in low-income countries.

